# Supplementary material for: End Sequence Analysis Toolkit (ESAT) expands the extractable information from single-cell RNA-seq data
Source: Genome Res. 2016 Oct;26(10):1397–410. doi: 10.1101/gr.207902.116 (PMC5052061; doi:10.1101/gr.207902.116)
Supplement: Supplemental Material [file supp_gr.207902.116_Supp_Materials.tar.gz › ESAT-esat_v0.1/doc/html/interfaceumms_1_1core_1_1annotation_1_1_annotation_collection_3_01_t_01extends_01_annotation_01_4.html]

ESAT: umms.core.annotation.AnnotationCollection< T extends Annotation > Interface Reference


ESAT


- Main Page
- Related Pages
- Packages
- Classes
- Files

- Class List
- Class Index
- Class Hierarchy
- Class Members

All Classes Namespaces Files Functions Variables Enumerator Pages

- umms
- core
- annotation
- AnnotationCollection< T extends Annotation >

Public Member Functions |
List of all members

umms.core.annotation.AnnotationCollection< T extends Annotation > Interface Reference

|  |  |
| --- | --- |
| Public Member Functions | |
| int | size () |
|  | |
| double | getGlobalCount () |
|  | |
| double | getCount (Annotation region, boolean fullyContained) |
|  | |
| double | getCount (Annotation region) |
|  | |
| double | getCount (AnnotationList<?extends Annotation > set) |
|  | |
| double | getCount (AnnotationList<?extends Annotation > set, boolean fullyContained) |
|  | |
| int | getBasesCovered (Annotation region, boolean fullyContained) |
|  | |
| int | getBasesCovered (Annotation region) |
|  | |
| double | getTotalCoverage (Annotation region) |
|  | |
| double | getAverageCoverage (Annotation region) |
|  | |
| Annotation | collapse (Annotation region, boolean fullyContained) |
|  | |
| CloseableIterator< T > | getOverlappingAnnotations (Annotation region) |
|  | |
| CloseableIterator< T > | getOverlappingAnnotations (Annotation region, boolean fullyContained) |
|  | |
| CloseableIterator< T > | iterator () |
|  | |
| void | addFilter (Predicate< T > filter) |
|  | |
| void | addFilters (Collection< Predicate< T >> filters) |
|  | |
| CloseableIterator< T > | getPermutedAnnotations (Annotation region) |
|  | |
| double | getCountExcludingRegion (Annotation region, Annotation excluded) |
|  | |
| double | getCountStrandedExcludingRegion (Annotation region, Annotation excluded) |
|  | |
| CoordinateSpace | getCoordinateSpace () |
|  | |
| WindowScoreIterator< CountScore > | scan (int windowSize, int overlap) |
|  | |
| public< W extends WindowScore >  WindowScoreIterator< W > | scan (int windowSize, int overlap, WindowProcessor< W > processor) |
|  | |
| public< W extends WindowScore >  WindowScoreIterator< W > | scan (Annotation region, int windowSize, int overlap, WindowProcessor< W > processor) |
|  | |
| WindowScoreIterator< CountScore > | scan (Annotation region, int windowSize, int overlap) |
|  | |
| double | getRefSequenceLambda (String refname) |
|  | |

## Detailed Description

Author
:   engreitz Interface to allow for counting over both AnnotationReader and ReadCollection.

## Member Function Documentation

|  |  |  |  |  |  |
| --- | --- | --- | --- | --- | --- |
| void umms.core.annotation.AnnotationCollection< T extends Annotation >.addFilter | ( | Predicate< T > | *filter* | ) |  |

Adds a filter to use to decide whether an alignment is valid

Parameters
:   |  |  |
    | --- | --- |
    | filter | the filter to use |

|  |  |  |  |  |  |
| --- | --- | --- | --- | --- | --- |
| void umms.core.annotation.AnnotationCollection< T extends Annotation >.addFilters | ( | Collection< Predicate< T >> | *filters* | ) |  |

Add a set of filters to decide whether an alignment is valid

Parameters
:   |  |  |
    | --- | --- |
    | filters | Collection of filters to add |

|  |  |  |  |
| --- | --- | --- | --- |
| Annotation umms.core.annotation.AnnotationCollection< T extends Annotation >.collapse | ( | Annotation | *region*, |
|  |  | boolean | *fullyContained* |
|  | ) |  |  |

Collapse all reads within the defined region into the overlapping set

Parameters
:   |  |  |
    | --- | --- |
    | region | The region to determine overlap |
    | fullyContained | Whether to count only fully contained reads |

Returns
:   null if there are no Annotations that overlap

|  |  |  |  |  |  |
| --- | --- | --- | --- | --- | --- |
| double umms.core.annotation.AnnotationCollection< T extends Annotation >.getAverageCoverage | ( | Annotation | *region* | ) |  |

Returns the average base-level coverage for the given region.

Parameters
:   |  |  |
    | --- | --- |
    | region |  |

Returns

|  |  |  |  |
| --- | --- | --- | --- |
| int umms.core.annotation.AnnotationCollection< T extends Annotation >.getBasesCovered | ( | Annotation | *region*, |
|  |  | boolean | *fullyContained* |
|  | ) |  |  |

This method will return the total number of bases that are covered by alignments in the collection If discontinuous region, it will only count blocks The maximum possible value is defined by region.getTranscriptSize();

Parameters
:   |  |  |
    | --- | --- |
    | region | The region to determine overlap |
    | fullyContained | Whether to count only fully contained reads |

Returns

|  |  |  |  |  |  |
| --- | --- | --- | --- | --- | --- |
| int umms.core.annotation.AnnotationCollection< T extends Annotation >.getBasesCovered | ( | Annotation | *region* | ) |  |

This method will return the total number of bases that are covered by alignments in the collection If discontinuous region, it will only count blocks. The maximum possible value is defined by region.getTranscriptSize(); Annotations counted here do not need to be fully contained in the region.

Parameters
:   |  |  |
    | --- | --- |
    | region | The region to determine overlap |

Returns

|  |  |  |  |  |
| --- | --- | --- | --- | --- |
| CoordinateSpace umms.core.annotation.AnnotationCollection< T extends Annotation >.getCoordinateSpace | ( |  | ) |  |

Returns
:   The coordinate space currently used by the AnnotationCollection

|  |  |  |  |
| --- | --- | --- | --- |
| double umms.core.annotation.AnnotationCollection< T extends Annotation >.getCount | ( | Annotation | *region*, |
|  |  | boolean | *fullyContained* |
|  | ) |  |  |

This method will return the number of reads overlapping a given region

Parameters
:   |  |  |
    | --- | --- |
    | region | The reference region to count overlapping elements |
    | fullyContained | whether to only count overlapping elements if they are fully contained within the reference region |

Returns
:   returns a double to allow implementing methods to utilize a weight object

Exceptions
:   |  |  |
    | --- | --- |
    | IOException |  |

|  |  |  |  |  |  |
| --- | --- | --- | --- | --- | --- |
| double umms.core.annotation.AnnotationCollection< T extends Annotation >.getCount | ( | Annotation | *region* | ) |  |

This method will return the number of reads overlapping a given region (not necessarily fully contained)

Parameters
:   |  |  |
    | --- | --- |
    | region | The reference region to count overlapping elements |

Returns
:   returns a double to allow implementing methods to utilize a weight object

Exceptions
:   |  |  |
    | --- | --- |
    | IOException |  |

|  |  |  |  |  |  |
| --- | --- | --- | --- | --- | --- |
| double umms.core.annotation.AnnotationCollection< T extends Annotation >.getCount | ( | AnnotationList<?extends Annotation > | *set* | ) |  |

|  |  |  |  |
| --- | --- | --- | --- |
| double umms.core.annotation.AnnotationCollection< T extends Annotation >.getCount | ( | AnnotationList<?extends Annotation > | *set*, |
|  |  | boolean | *fullyContained* |
|  | ) |  |  |

|  |  |  |  |
| --- | --- | --- | --- |
| double umms.core.annotation.AnnotationCollection< T extends Annotation >.getCountExcludingRegion | ( | Annotation | *region*, |
|  |  | Annotation | *excluded* |
|  | ) |  |  |

Compute the reads fully contained in region that are NOT overlapping excluded

Parameters
:   |  |  |
    | --- | --- |
    | region |  |
    | excluded |  |

Returns
:   Count contained in region that do not overlap the excluded region

|  |  |  |  |
| --- | --- | --- | --- |
| double umms.core.annotation.AnnotationCollection< T extends Annotation >.getCountStrandedExcludingRegion | ( | Annotation | *region*, |
|  |  | Annotation | *excluded* |
|  | ) |  |  |

|  |  |  |  |  |
| --- | --- | --- | --- | --- |
| double umms.core.annotation.AnnotationCollection< T extends Annotation >.getGlobalCount | ( |  | ) |  |

Returns
:   Global count, possibly weighted. Might be different from size if only counting reads that map, etc.

|  |  |  |  |  |  |
| --- | --- | --- | --- | --- | --- |
| CloseableIterator<T> umms.core.annotation.AnnotationCollection< T extends Annotation >.getOverlappingAnnotations | ( | Annotation | *region* | ) |  |

This method will return a collection of the annotations that overlap a given region (not necessarily fully contained)

Parameters
:   |  |  |
    | --- | --- |
    | region | The region to overlap |

Returns

|  |  |  |  |
| --- | --- | --- | --- |
| CloseableIterator<T> umms.core.annotation.AnnotationCollection< T extends Annotation >.getOverlappingAnnotations | ( | Annotation | *region*, |
|  |  | boolean | *fullyContained* |
|  | ) |  |  |

This method will return a collection of the annotations that overlap a given region

Parameters
:   |  |  |
    | --- | --- |
    | region | The region to determine overlap |
    | fullyContained | Whether to count only fully contained reads |

Returns

|  |  |  |  |  |  |
| --- | --- | --- | --- | --- | --- |
| CloseableIterator<T> umms.core.annotation.AnnotationCollection< T extends Annotation >.getPermutedAnnotations | ( | Annotation | *region* | ) |  |

Shuffle the locations of annotations within the given region and return an iterator over the permuted annotations. Permuted annotations will not be necessarily sorted. NOTE: This will alter the annotations in the reader, so re-reading or reloading might be necessary.

Parameters
:   |  |  |
    | --- | --- |
    | region | reads reads fully contained in this region will be shuffled |

|  |  |  |  |  |  |
| --- | --- | --- | --- | --- | --- |
| double umms.core.annotation.AnnotationCollection< T extends Annotation >.getRefSequenceLambda | ( | String | *refname* | ) |  |

|  |  |  |  |  |  |
| --- | --- | --- | --- | --- | --- |
| double umms.core.annotation.AnnotationCollection< T extends Annotation >.getTotalCoverage | ( | Annotation | *region* | ) |  |

Returns the total base-level coverage for the given region.

Parameters
:   |  |  |
    | --- | --- |
    | region |  |

Returns

|  |  |  |  |  |
| --- | --- | --- | --- | --- |
| CloseableIterator<T> umms.core.annotation.AnnotationCollection< T extends Annotation >.iterator | ( |  | ) |  |

Returns
:   A closeable iterator through all annotations that pass the filters.

|  |  |  |  |
| --- | --- | --- | --- |
| WindowScoreIterator<CountScore> umms.core.annotation.AnnotationCollection< T extends Annotation >.scan | ( | int | *windowSize*, |
|  |  | int | *overlap* |
|  | ) |  |  |

|  |  |  |  |
| --- | --- | --- | --- |
| public<W extends WindowScore> WindowScoreIterator<W> umms.core.annotation.AnnotationCollection< T extends Annotation >.scan | ( | int | *windowSize*, |
|  |  | int | *overlap*, |
|  |  | WindowProcessor< W > | *processor* |
|  | ) |  |  |

|  |  |  |  |
| --- | --- | --- | --- |
| public<W extends WindowScore> WindowScoreIterator<W> umms.core.annotation.AnnotationCollection< T extends Annotation >.scan | ( | Annotation | *region*, |
|  |  | int | *windowSize*, |
|  |  | int | *overlap*, |
|  |  | WindowProcessor< W > | *processor* |
|  | ) |  |  |

|  |  |  |  |
| --- | --- | --- | --- |
| WindowScoreIterator<CountScore> umms.core.annotation.AnnotationCollection< T extends Annotation >.scan | ( | Annotation | *region*, |
|  |  | int | *windowSize*, |
|  |  | int | *overlap* |
|  | ) |  |  |

|  |  |  |  |  |
| --- | --- | --- | --- | --- |
| int umms.core.annotation.AnnotationCollection< T extends Annotation >.size | ( |  | ) |  |

Returns
:   Total number of annotations. Considers filters.

---

The documentation for this interface was generated from the following file:

- java/umms/core/annotation/AnnotationCollection.java


---

Generated on Fri Aug 15 2014 15:11:08 for ESAT by  

 1.8.7
